# Magnitude and Predisposing Factors of Difficult Airway during Induction of General Anaesthesia

**DOI:** 10.1155/2017/5836397

**Published:** 2017-07-11

**Authors:** Sileshi Abiy Workeneh, Amare Hailekiros Gebregzi, Zewditu Abdissa Denu

**Affiliations:** College of Medicine and Health Science, Department of Anesthesiology and Critical Care, University of Gondar, Gondar, Ethiopia

## Abstract

**Objective:**

To assess magnitude and predisposing factors of difficult airway during induction of general anaesthesia.

**Methods:**

Hospital based cross sectional study carried out to determine the incidence of difficult mask ventilation, difficult laryngoscopy (Cormack and Lehane III and IV), difficult intubation (IDS ≥ 5), and failed intubation. The association between each predisposing factor and airway parameters with components of difficult airway is investigated with binary logistic regression. Sensitivity, specificity, positive and negative predictive value of the test, and odds ratio with 95% confidence interval were calculated to determine the association between independent and dependent variable.

**Result:**

The incidence of difficult laryngoscopy, difficult intubation, and failed intubation are 12.3%, 9%, and 0.005%, respectively. Mouth opening < 30 mm and Mallampati classes III and IV are the most sensitive tests and second high specific test next to combination of tests to predict difficult intubation and laryngoscopy (*P* value < 0.001). Unrestricted multiple attempt without alternative airway techniques resulted in exponential increase in desaturation episodes and further difficulty of airway management (*P* value < 0.001).

**Discussion and Conclusion:**

Mallampati classes III and IV, mouth opening ≤ 30 mm, jaw slide grade C, attempt > 3, and ineffective alternative technique have increased predictability value of difficult airway.

## 1. Introduction

It is widely accepted that general anaesthesia is not without morbidity. One of the well-known life threatening events associated with general anaesthesia is difficult airway which can happen during induction of anaesthesia while attempting to insert the endotracheal tube with the aid of laryngoscope [1].

According to the American Society of Anaesthesiologists (ASA) difficult airway is defined as the situation in which the “conventionally trained anaesthesiologist experiences difficulty with intubation, mask ventilation, or both” [2].

This study will focus on creating awareness about the extent of problems and predominant risk factors related to difficult airway management. It also addresses gaps in current practice and tries to forward solutions comparable to the standards/guidelines practiced worldwide which can be suitable and specific to Gondar University Hospital (GUH) setup.

The size and distribution of this problem are not well known or documented in GUH. It is only reported if complications occur, either in critical incident meetings or during litigation.

The complications related to poor/inappropriate management of difficult airway are death, brain damage, ICU admission, prolonged recovery, emergency surgical airway, and trauma to airway and teeth which require high level care and extra cost [3–6].

The laryngoscopic view Cormack and Lehane grade 3 and 4 incidence is also inconsistent among recently done studies which shows figures ranges 3% to 16% and also can go higher in patients with goitre like 23 out of 80 patients [4, 7].

One prospective study done in Turkey showed the incidence of difficult intubation as 4.8% which is more similar to other studies done in other different countries [8]. But it is severalfold increased to 1 in 17 and around 13.8% in emergency obstetric and obese patients, respectively [3, 9].

The studies done previously concentrated and focused on identifying parameters/test that can predict difficult airway. The risk factors or airway parameters for DMV, DL, and DI like jaw slide test, pillow height, and Mallampati score are among the parameters widely researched. The results of this studies are inconsistent with each other which is mainly altered by patient characteristics, definition variations, type of study mainly retrospective versus prospective, and underreport of many cases due to compliance and litigation issues. There are limited studies done about risk factors involving some of those airway parameters like poor preoperative airway evaluation, not having predetermined plan, poor clinical decisions, unfamiliarity for airway events, and availability of equipment [1–4, 7, 9, 10].

The practice of combining airway parameters or risk factors to predict anticipated difficult ventilation and intubation may appear self-evident; however, it has not been rigorously evaluated and results are also conflicting [1, 8]. So this study investigates whether combination or single airway parameters are preferred and are evidence based clinical practice to predict difficult airway. The aim of this study is to determine the magnitude and predisposing factors of difficult airway during induction of general anaesthesia.

## 2. Methodology

The study was done after obtaining an agreement letter for approval by institutional ethics committee to investigate this problem.

Prospective observational study was done in GUH operation theatres located in Gondar town, northwest Ethiopia from 01 March to 20 April 2015. This hospital is tertiary teaching hospital which gives operation service for more than 3 million people living in Gondar city and surroundings. All surgical patients were operated on at GUH with GA and ETT in the study period.

Preoperative airway assessment parameters like Mallampati class, mouth opening or interincisors gap, and other independent variables which are alternative technique, number of attempts, and desaturation episode {SpO_2_ < 95%} were used to predict components of difficult airway. Difficult laryngoscopy (Cormack and Lehane's grades III and IV) and difficult intubation also defined as intubation difficulty scale (IDS) > 5 was taken as dependent variables. All patients operated on in the specified study period were included.

A pretested and structured questionnaire was prepared to collect data from the patient a day before surgery and during induction of anaesthesia. Independent variables like airway parameters were collected by observing and measuring each airway assessment test. The data for dependent variable was collected during induction by qualified duty free anaesthetist by observation. Data about the grade of laryngoscopy were collected from anaesthetic record sheet. Two qualified anaesthetists for data collection were selected based on the capability of being free during the data collection period and experience of data collection.

By using SPSS 20 version statistical package analysis was done. Odds ratios with 95% of confidence interval, sensitivity, specificity, and positive and negative predictive value were calculated to assess the association between the outcome and exposure variables. Binary logistic regression was used to assess the influence of each and combination of risk factors or airway parameters on the incidence of difficult laryngoscopy and difficult intubation.

## 3. Result

Data from 212 patients were evaluated (Table 1). The incidence of difficult laryngoscopy (Cormack and Lehane grades III & IV) difficult intubation is 12.3%, and 9%, respectively, with no patient having difficult mask ventilation.

Table 2 shows the incidence of predisposing factors for a difficult airway. Oropharyngeal view, jaw slide grade, and mouth opening/interincisor gap are statistically significant to predict difficult airway with *P* value less than 0.05.

### 3.1. Difficult Laryngoscopy and Difficult Airway Predictors

The sensitivity, specificity, predictive value of positive test {PVPT}, and predictive value of the negative test {PVNT} of each airway parameter and difficult laryngoscopy are shown in Table 4.

**Figure 1:**
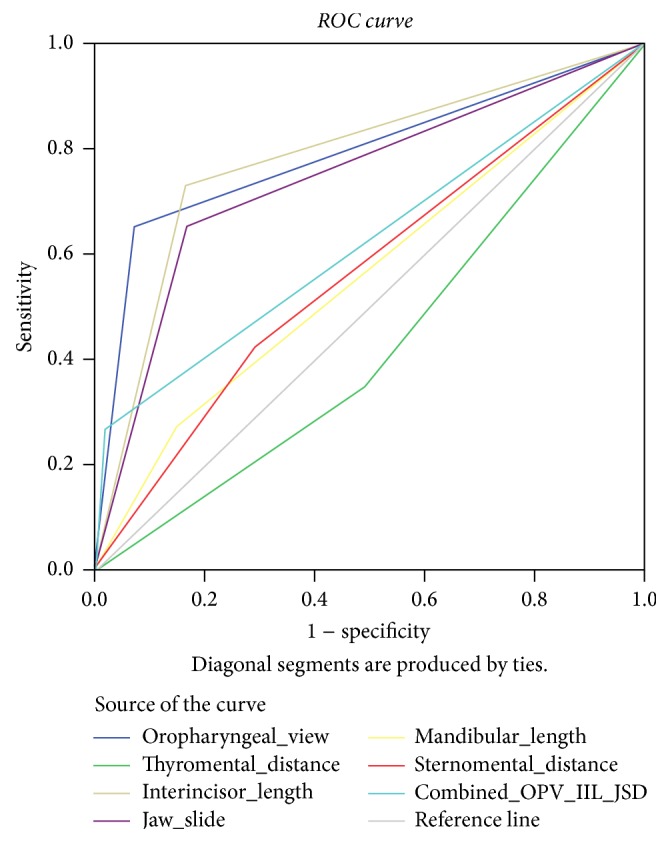
ROC curve of difficult laryngoscopy and airway parameters.

The actual figures of Table 4 used to produce the ROC curve. Both Table 4 and Figure 1 demonstrate that OPV, IIL, and JSD are all seen to be highly significant in predicting difficult laryngoscopy.

The ROC curve in Figure 1 shows all airway screening tests are above the reference line except thyromental length {TMD}. The other 3 tests, that is, OPV, IIL, and JSD, are more consistent to sensitivity and 1 − specificity line and also with an increased area under the curve compared to the combined and the remaining airway tests.

### 3.2. Difficult Intubation and Difficult Airway Predictors

The sensitivity, specificity, predictive value of positive test {PVPT}, and predictive value of the negative test {PVNT} of each airway parameter and difficult laryngoscopy are shown in Table 5.

**Figure 2:**
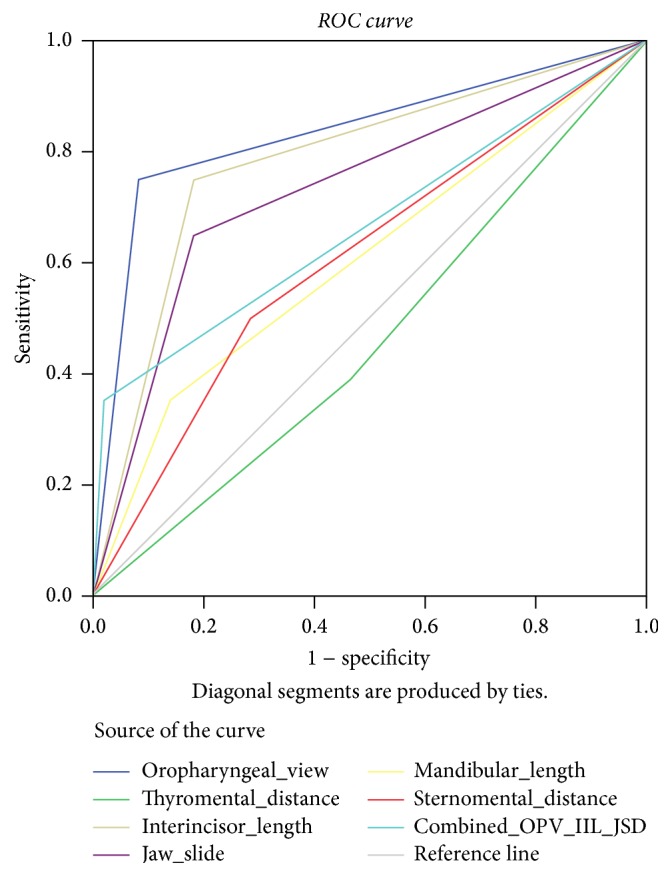
ROC curve of difficult intubation and airway parameters.

The Mallampati classes III & IV, mouth opening < 30 mm, and jaw slide grade C are the airway parameters found to the most sensitive tests {75%, 75%, and 65%, resp.}. Also like difficult laryngoscopy, thyromental distance fails to show predictability of difficult intubation (Figure 2).

Unlike difficult laryngoscopy, OPV class is the most sensitive test followed by mouth opening and jaw slide as represented in Figures 1 and 2.

### 3.3. Other Infrequent Predisposing Risk Factors and Outcome of Difficult Intubation

Table 6 showed the incidence of difficult intubation and laryngoscopy tends to increase in patients with repeated attempts with limited strategies to improve laryngoscopic visualization and insertion of the ETT.

## 4. Discussion

The incidence of difficult laryngoscopy, difficult intubation, and failed intubation is 12.3%, 9%, and 0.47%, respectively, with no patient having difficult mask ventilation. Even though the overall incidence of difficult laryngoscope and intubation in this study was found to be on the upper border of the usual figures mentioned in most papers, it is still consistent with studies done in similar environment, patients, and level of competency of anaesthetists, anaesthesia providers, and trainers in tertiary teaching hospitals [11, 12].

The incidence of difficult intubation in a similar study done in Dublin, Ireland, with the same sample size was 9% [13]. Another study done in France on a large number of goitre patients which is also the most dominant procedure done in GUH demonstrated the incidence of difficult laryngoscopy and intubation to be 10%  and  8%, respectively, which is also consistent with this study [7]. The incidence of difficult intubation in the study on 3,423 emergency tracheal intubations at university hospital with trainers in Michigan USA was 10.8%, a relatively increased percentage of complication which is consistent since most intubations done in GUH are by trainers [12]. In the comparative study on direct laryngoscopic views depending on pillow height the incidence of difficult laryngoscopy (Cormack and Lehane grade 3) was 16% without a pillow which is a similar practice to that GUH operation room [14]. The incidence of difficult intubation in the study done on the incidence and associated risk factors of difficult mask ventilation in Turkey demonstrated 21.3% [11].

There is still no single test with 100% sensitivity and specificity to predict difficult laryngoscopy and intubation. Among airway parameters, Mallampati classifications III & IV {sensitivity 65%, specificity 92.5%}, mouth opening ≤ 30 mm {sensitivity 73.3%, specificity 83.1%}, and jaw slide grade C were found to be a balanced measure of sensitivity, specificity, PVPT, and PVNT in this study. These tests have high level of significance compared to others {*P* value < 0.001} for both difficult laryngoscope and intubation {Tables 3 and 4}. Previously done studies showed that airway screening tests are more capable of ruling out patients without risk of difficult airway than predicting difficulty. There is a wide variety of Mallampati classification sensitivity figures like 35%, 44%, and 87.5% in studies done in Turkish patients {*P* value < 0.05}, in preoperative assessment test study in USA, and in Nigerian obstetric patient, respectively. In contrast to this, but in keeping with GUH results, there is consistency in Mallampati class and mouth opening specificity results ranging from 80% to 95% for both difficult intubation and laryngoscopy in most studies [8, 15, 16]. In one double blinded control trial study and another prospective study done on extensive number of patients on mouth opening < 30 and 40 mm, respectively, a strong association with difficult laryngoscopy and intubation with a high level of significance was showed {*P* value < 0.05} [6, 7]. Mouth opening <30 mm was also found to be statistically significant {*P* value < 0.05} in study done by Mallat et al. on prediction of difficult intubation [7, 8].

The specificity and positive predictive value of the combined test for difficult laryngoscopy and intubation are greater than specificity and PPV of each single test in this study {Table 3}, but the ability to identify difficult laryngoscopy and intubation cases {sensitivity = 26.9%/35%} is the least compared to others. So by combining all the tests, there is an increased possibility of identifying those patients who did not face difficult laryngoscopy and intubation {increased specificity}, but not being able to increase the possibility of detecting difficult airway cases {sensitivity} compared to isolated Mallampati class and mouth opening. The combined airway screening test on study done on prediction of difficult laryngoscopy in emergency caesarean section failed to show higher detection of obstetric subjects with difficulty in laryngoscopy and intubation than other single screening tests {sensitivity 0.21, *P* value = 0.2} [3]. A multicentre methodological study demonstrated combining tests did not improve those results of single screening test [8].

**Figure 3:**
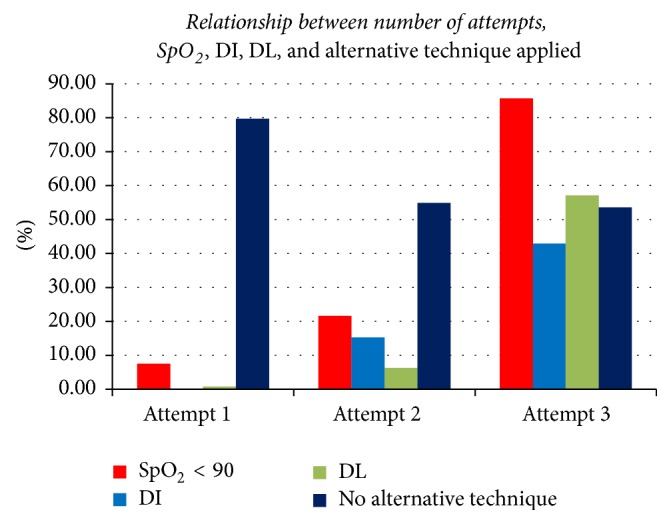
Relationship between number of attempts, SpO_2_, DI, DL, and alternative technique applied.

Unrestricted multiple attempts of direct laryngoscopy often result in trauma to the airway structures which leads to bleeding and oedema that potentially interfere with visualization of the cords {Table 5}. Prolonged periods of apnoea, dysrhythmias especially bradycardia, and need of additional anaesthetic drugs due to prolonged and repeated attempts of airway laryngoscopy are also reported in the studies [17–19]. In this study as shown in Table 5, repeated attempts of laryngoscopy had shown causative relationship to predominant adverse event occurred, that is, desaturation {SpO_2_ < 90%}. Even though multiple attempts can cause difficult airway and vice versa, the incidence of difficult laryngoscopy occurred after 3 attempts were increased 4 times compared to attempts less than 2 times {*P* value = 0.01, Table 5 and Figure 3}. This study also shows, with an increased number of attempts, there was exponential increase in the incidence of difficult intubation {Ex (B) = 5.47, *P* value = 0.042}, failed alternative technique, and desaturation in the followed subsequent attempts simultaneously. Two studies done in USA showed that attempts above 3 attempts are associated with 5.3–13.9 times increased incidence of difficult laryngoscopy and intubation and 2 times increased failed intubation which leads to severe adverse events [20, 21]. The NAP4 also reported that multiple attempts {>5 attempts} in 5 patients out of 41 were a leading and frequent cause of difficult airway and ICU admission secondary to airway trauma. “It is therefore important to assure that the first attempt at laryngoscopy is a best attempt” [17].

Desaturation episodes {SpO_2_ < 90%} did not go further to serious harmful adverse events or death in all complicated patients (Figure 3). But the desaturation episodes are 7 times greater in patients with difficult airway than patients with easy intubation {*P* value < 0.001} (Table 5). This figure can be comparative and similar for airway management ending up with failed intubation, death, or retrospective case studies from closed claims [22]. The increased incidence of desaturation episodes during airway instrumentation without difficult mask ventilation can be explained with inadequate maximum alveolar oxygen store during preoxygenation due to a loose-fitting mask, allowing the entrainment of room air, prolonged time of laryngoscopy, and intubation or underestimation of the risk which means negligence in this scenario [17].

Patients whose attempt of laryngoscopy was done above 3 {*N* = 28} and 53.6%  {*N* = 15} did not get any alternative technique to minimize or succeed the next attempt/attempts. Most of these patients desaturate and became difficult to intubate in the next unrestricted attempts. So those patients who were difficult in the first attempt did not a promising second alternative technique regarding either prevention of hypoxia or alternative technique which improves laryngoscopic view and enables successful intubation in subsequent attempts. So the subsequent attempt was not even good enough compared to previous attempt in managing difficult laryngoscopic, intubation, and oxygen desaturation. Successful alternative techniques like using a boogie or giving priority to ventilation rather than continuing with airway instrumentation are not practiced especially during difficult airway cases. The other well-known techniques to improve laryngeal view are that external laryngeal manipulation by the laryngoscopist or trained assistant which is proved to improve more than 13–15% of Cormack and Lehane III & IV grade in studies done on goitre and emergency patients [7, 23].

In conclusion, the incidence of DL, DI, and FI is 12.3%, 9%, and 0.47% in GUH surgical patients. The most important predominant risk factors observed are Mallampati classes III & IV, mouth opening < 30 mm, JSD grade C, attempt > 3, and ineffective alternative technique. A combination of bedside airway parameters to predict difficult airway is not evidence based clinical practice since more than 60% difficult airway cases missed. Since all difficult airway cannot be anticipated before induction, high index of suspicion and adequate preparation with predetermined plan should be practiced.

## Figures and Tables

**Table 1:** Patient demographics.

Variable	Frequency *n* {%}
Age	
Under 16	42 {19.8%}
17–50	153 {72.2%}
Above 51	17 {8%}
Sex	
Male	99 {46.7%}
Female	113 {53.4%}
BMI	
Underweight	22 {10.4%}
Normal	141 {66.5%}
Overweight	10 {4.7%}
Obese	3 {1.4%}
Type of surgery	
Elective	80 {37.7}
Emergency	132 {62.3%}
Surgical specialty	
ENT surgeries	15 {7.1%}
Obstetrics-gynecology	58 {27.4%}
General surgery	111 {52.4%}
Other	28 {13.2%}

**Table 2:** Airway parameters and risk factors, ^*∗*^*P* value < 0.05.

Variable	Frequency *n* {%}
Oropharyngeal view (OPV)	
OPV I & II	150 {70.8%}
OPV III & IV	31 {14.6%}^**∗**^
Jaw slide grade (JSD)	
JSD A	73 {34.4%}
JSD B	91 {42.9%}
JSD C	17 {8%}^**∗**^
Mouth opening (interincisor length)	
<30 mm	18 {8.9%}
31–50 mm	149 {70.3%}
>51 mm	13 {6.1%}^**∗**^
Mandibular length (MBL)	
<70 mm	4 {1.9%}
71–85 mm	30 {14.2%}
86–95 mm	80 {37.7%}
>96 mm	67 {31.6}
Sternomental distance (SMD)	
81 mm–120 mm	65 {30.7%}
121 mm–135 mm	76 {35.6%}
>136 mm	40 {18.9}
Thyromental distance (TMD)	
<50 mm	2 {0.9%}
51–65 mm	67 {31.6%}
66–75 mm	67 {31.6%}
>76 mm	45 {21.2%}

**Table 3:** Airway management techniques and their outcome.

Variable	Frequency *n* {%}
Attempt	
1 attempt	133 {62.7%}
2 attempts	51 {24.1%}
≥3 attempts	28 {13.2%}^**∗**^
Alternative technique	
Applied	63 {29.7%}
Not applied	149 {70.3%}
Laryngeal manipulation	
Applied	129 {60.8%}
Not applied	83 {39.2%}
Desaturation (SpO_2_ < 90%) episodes	45 {21.2%}^**∗**^

*∗* signifies statistical significance.

**Table 4:** Difficult laryngoscopy and difficult airway predictors.

Variables	Sensitivity	Specificity	PVPT	PVNT	Area	*P* value	95% CI interval	
							Upper	Lower
OPV	65.4%	92.5%	54.8%	95.0%	0.789	<0.001	0.677	0.902
JSD	65.4	83.3%	35.4%	94.5%	0.744	<0.001	0.631	0.856
ILL	73.1%	83.3%	58%	95.7%	0.788	<0.001	0.678	0.886
Combined	26.9%	97.8%	63.6%	90.5%	0.624	0.041	0.494	0.754
SMD	42.3%	71%	16.9%	84.8%	0.566	0.273	0.445	0.687
MBL	26.9%	85.5%	20.6%	85.5%	0.562	0.306	0.438	0.686
TMD	34.6%	51.1%	9%	84.5%	0.428	0.238	0.313	0.544

**Table 5:** Difficult intubation and difficult airway predictors.

Variables	Sensitivity	Specificity	PVPT	PVNT	Area	*P* value	95% CI interval	
							Upper	Lower
OPV	75%	91.7%	48.4%	97.2%	0.834	<0.001	0.719	0.948
JSD	65%	81.8%	27.1%	95.7%	0.734	0.001	0.607	0.861
ILL	75%	81.8%	30%	96.9%	0.784	<0.001	0.669	0.898
Combined	35%	97.9%	63.6%	97.9%	0.665	0.015	0.517	0.812
SMD	50%	71.4%	15.4%	93.2%	0.607	0.124	0.472	0.742
MBL	35%	85.9%	20.6%	92.7%	0.605	0.116	0.463	0.764
TMD	40%	52.1%	8%	89.3%	0.460	0.560	0.329	0.592

**Table 6:** Risk factors, management carried out, and its outcome.

Variable	*P* value DL/DI	Odds r. DL/DI	95% CI DL/DI	
			Lower	Upper
Attempt {≥3}	0.010/0.042	4.031/5.47	1.393/2.32	11.669/16.02
Laryngeal manipulation	0.13/0.13	4.061/6.568	1.346/1.482	12.252/29.103
Desaturation episodes {SpO_2_ < 90%}	<0.001/0.01	7.227/7.091	2.74/2.969	19.065/16.936

PA and PP: partially available and partially prepared.
